# 2-(Benzoyl­amino­meth­yl)pyridinium chloride

**DOI:** 10.1107/S1600536808037021

**Published:** 2008-11-13

**Authors:** Christian Koch, Helmar Görls, Matthias Westerhausen

**Affiliations:** a Institute of Inorganic and Analytical Chemistry, Friedrich-Schiller-Universität Jena, August-Bebel-Strasse 2, D-07743 Jena, Germany

## Abstract

The title compound, C_13_H_13_N_2_O^+^·Cl^−^, (1), was obtained as a colorless crystalline by-product during the synthesis of *N*-(2-pyridylmeth­yl)benzoyl­amine (2). The C—O bond length of 1.231 (2) Å in the benzoyl unit of (1) is slightly elongated in comparison with isolated C=O double bonds as also observed for (2) [1.237 (2) Å]. The N—C bond length of 1.345 (2) Å in the benzoic acid amide unit indicates the formation of an allylic O—C—N system and is very similar to the N—C bond lengths [1.345 (2) Å] of the pyridyl group. A further delocalization of charge from this allylic system into the phenyl fragment does not occur, which can be deduced from a characterisitc C—C single bond length of 1.499 (2) Å between these fragments. A dimer is formed *via* N—H⋯Cl hydrogen bonds. The two rings make a dihedral angle of 105.0 (2)°

## Related literature

For general background, see: Westerhausen *et al.* (2001 ▶, 2002 ▶). For related structures, see: Koch *et al.* (2008 ▶); Prostota *et al.* (2004 ▶).
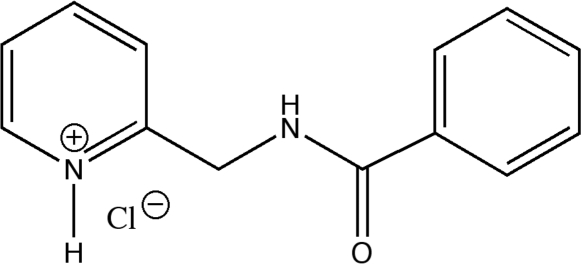


## Experimental

### 

#### Crystal data

C_13_H_13_N_2_O^+^·Cl^−^
*M* *_r_* = 248.70
Monoclinic, 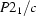

*a* = 4.6159 (1) Å
*b* = 27.4573 (10) Å
*c* = 9.6851 (4) Å
β = 96.554 (2)°
*V* = 1219.47 (7) Å^3^
*Z* = 4
Mo *K*α radiation
μ = 0.30 mm^−1^
*T* = 183 (2) K
0.05 × 0.05 × 0.05 mm

#### Data collection

Nonius KappaCCD diffractometer
Absorption correction: none
7630 measured reflections
2776 independent reflections
2094 reflections with *I* > 2σ(*I*)
*R*_int_ = 0.036

#### Refinement

*R*[*F* ^2^ > 2σ(*F* ^2^)] = 0.037
*wR*(*F* ^2^) = 0.095
*S* = 1.00
2776 reflections
206 parameters
All H-atom parameters refined
Δρ_max_ = 0.20 e Å^−3^
Δρ_min_ = −0.25 e Å^−3^

### 

Data collection: *COLLECT* (Nonius, 1998 ▶); cell refinement: *DENZO* (Otwinowski & Minor, 1997 ▶); data reduction: *DENZO*; program(s) used to solve structure: *SHELXS97* (Sheldrick, 2008 ▶); program(s) used to refine structure: *SHELXL97* (Sheldrick, 2008 ▶); molecular graphics: *SHELXTL/PC* (Sheldrick, 2008 ▶); software used to prepare material for publication: *SHELXL97*.

## Supplementary Material

Crystal structure: contains datablocks I, global. DOI: 10.1107/S1600536808037021/dn2394sup1.cif

Structure factors: contains datablocks I. DOI: 10.1107/S1600536808037021/dn2394Isup2.hkl

Additional supplementary materials:  crystallographic information; 3D view; checkCIF report

## Figures and Tables

**Table 1:** Hydrogen-bond geometry (Å, °)

*D*—H⋯*A*	*D*—H	H⋯*A*	*D*⋯*A*	*D*—H⋯*A*
N1—H1*N*1⋯Cl1	0.86 (2)	2.41 (2)	3.2057 (15)	153.3 (18)
N2—H1*N*2⋯Cl1^i^	0.93 (2)	2.13 (2)	3.0446 (16)	171.7 (18)

Symmetry code: (i) 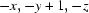.

**Fig. 1.**
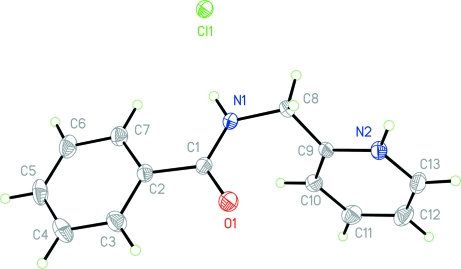
The molecular structure of the title compound with the atom-labeling scheme. Ellipsoids are drawn at the 40% probability level. H atoms are represented as smal spheres of arbitrary radii.

**Fig. 2.**
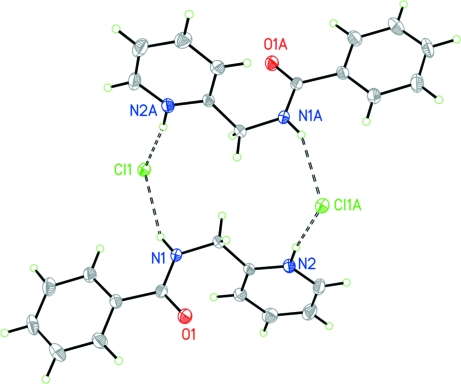
A view of the pseudo dimer formed by N-H···Cl hydrogen bonds shown as dashed lines. H atoms are represented as small spheres of arbitrary radii.[Symetry code: (A)1 - x, -y + 1, -z].

**Fig. 3.**
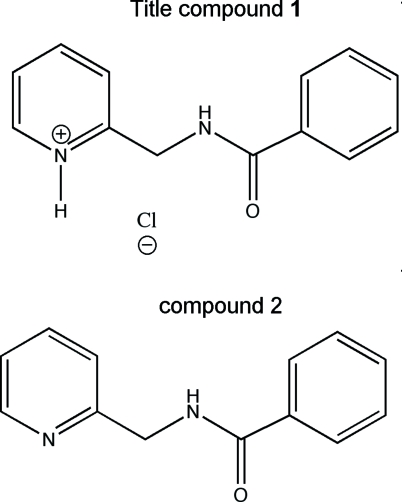
Compounds (1) and (2).

## supplementary crystallographic information

### Comment

In the past, metallated (2-pyridylmethyl)(trialkysilyl)amines were used for zinc-mediated oxidative C–C coupling reactions yielding [1,2-dipyridyl-1,2-bis(triisopropylsilylamido)ethane] bis(methylzinc) (Westerhausen *et al.* 2001 and 2002). The reaction of (2-pyridylmethyl)(*tert*-butyldimethylsilyl)amine and benzoyl chloride in toluene quantitatively yields *N*-(2-pyridylmethyl)benzoylamine ((1)) (Koch *et al.* 2008). Treatment of (1) with benzoyl chloride after deprotonation with butyllithium gives *N*-(2-pyridylmethyl)dibenzoylamine with rather poor yields. The title compound *N*-(2-pyridylmethyl)benzoylamine hydrochloride was also obtained as a colorless crystalline by-product.

A view of the title compound is shown in Fig. 1 . The C1—O1 bond length of 1.231 (2) Å is slightly elongated in comparison to isolated C=O double bonds. The value of the N1—C1 bond length of 1.345 (2)Å shows the formation of an allylic O1—C1—N1 system and is very similar to the N2—C9 bond length [1.345 (2) Å] of the pyridyl group. A further delocalization of charge from this allylic system into the phenyl fragment can be excluded on the basis of a characterisitc C8—C9 single bond of 1.499 (2) Å.

Two molecules are linked through N-H···Cl hydrogen bonds to form a pseudo dimer (Table 1, Fig. 2)

### Experimental

All manipulations were carried out in an atmosphere of argon using standard Schlenk techniques. Toluene and pentane were dried (Na/benzophenone) and distilled prior to use. 2-Pyridylmethylamine and butyllithium were purchased form Aldrich. Tert-butyldimethylchlorosilane and benzoyl chloride were purchased from Merck.^1^H NMR and ^13^C NMR spectra were recorded at [D_1_]chloroform solutions at ambient temperature on a Bruker AC 400 MHz s pectrometer and were referenced to deuterated benzene as an internal standard. ^1^H NMR (200 MHz, [D_1_]chloroform) *d* = 8.93 (s, br.,1*H*, NH); 8.63 (d, 1H, Pyr13); 8.35 (t, 1H, Pyr11); 8.06 (d, 1H, Pyr10); 7.98 (d, 2H, Ph); 7.79 (t, 1H, Pyr12); 7.48–7.37 (m, 3H, Ph); 5.03 (d, 2H, CH_2_)

### Refinement

All hydrogen atoms bonded were located by difference Fourier synthesis and freely refined.

### Crystal data

**Table:**

C_13_H_13_N_2_O^+^·Cl^–^	*F*_000_ = 520
*M**_r_* = 248.70	*D*_x_ = 1.355 Mg m^−^^3^
Monoclinic, *P*2_1_/*c*	Mo *K*α radiation λ = 0.71073 Å
Hall symbol: -P2ybc	Cell parameters from 7630 reflections
*a* = 4.61590 (10) Å	θ = 2.2–27.5º
*b* = 27.4573 (10) Å	µ = 0.30 mm^−^^1^
*c* = 9.6851 (4) Å	*T* = 183 (2) K
β = 96.554 (2)º	Prism, colourless
*V* = 1219.47 (7) Å^3^	0.05 × 0.05 × 0.05 mm
*Z* = 4	

### Data collection

**Table:**

Nonius KappaCCD diffractometer	2094 reflections with *I* > 2σ(*I*)
Radiation source: fine-focus sealed tube	*R*_int_ = 0.036
Monochromator: graphite	θ_max_ = 27.5º
*T* = 183(2) K	θ_min_ = 2.2º
φ and ω scans	*h* = −5→4
Absorption correction: none	*k* = −35→35
7630 measured reflections	*l* = −12→11
2776 independent reflections	

### Refinement

**Table:**

Refinement on *F*^2^	Secondary atom site location: difference Fourier map
Least-squares matrix: full	Hydrogen site location: inferred from neighbouring sites
*R*[*F*^2^ > 2σ(*F*^2^)] = 0.037	All H-atom parameters refined
*wR*(*F*^2^) = 0.095	*w* = 1/[σ^2^(*F*_o_^2^) + (0.0422*P*)^2^ + 0.4338*P*] where *P* = (*F*_o_^2^ + 2*F*_c_^2^)/3
*S* = 1.00	(Δ/σ)_max_ = 0.001
2776 reflections	Δρ_max_ = 0.21 e Å^−^^3^
206 parameters	Δρ_min_ = −0.25 e Å^−^^3^
Primary atom site location: structure-invariant direct methods	Extinction correction: none

### Special details

**Table:**

Geometry. All e.s.d.'s (except the e.s.d. in the dihedral angle between two l.s. planes) are estimated using the full covariance matrix. The cell e.s.d.'s are taken into account individually in the estimation of e.s.d.'s in distances, angles and torsion angles; correlations between e.s.d.'s in cell parameters are only used when they are defined by crystal symmetry. An approximate (isotropic) treatment of cell e.s.d.'s is used for estimating e.s.d.'s involving l.s. planes.
Refinement. Refinement of *F*^2^ against ALL reflections. The weighted *R*-factor *wR* and goodness of fit *S* are based on *F*^2^, conventional *R*-factors *R* are based on *F*, with *F* set to zero for negative *F*^2^. The threshold expression of *F*^2^ > σ(*F*^2^) is used only for calculating *R*-factors(gt) *etc*. and is not relevant to the choice of reflections for refinement. *R*-factors based on *F*^2^ are statistically about twice as large as those based on *F*, and *R*- factors based on ALL data will be even larger.

### Fractional atomic coordinates and isotropic or equivalent isotropic displacement parameters (Å^2^)

**Table:**

	*x*	*y*	*z*	*U*_iso_*/*U*_eq_	
O1	−0.2116 (3)	0.65339 (4)	−0.04007 (13)	0.0309 (3)	
N1	0.1330 (3)	0.59929 (5)	0.03755 (15)	0.0239 (3)	
H1N1	0.271 (5)	0.5898 (8)	0.099 (2)	0.042 (6)*	
N2	−0.1329 (3)	0.54981 (5)	−0.30377 (15)	0.0243 (3)	
H1N2	−0.255 (5)	0.5266 (7)	−0.272 (2)	0.039 (6)*	
C1	0.0007 (3)	0.64277 (6)	0.04314 (17)	0.0221 (3)	
C2	0.1261 (3)	0.67800 (6)	0.15220 (17)	0.0232 (4)	
C3	−0.0038 (4)	0.72351 (7)	0.1548 (2)	0.0330 (4)	
H3	−0.171 (5)	0.7308 (7)	0.087 (2)	0.043 (6)*	
C4	0.1007 (5)	0.75799 (7)	0.2522 (2)	0.0408 (5)	
H4	0.007 (5)	0.7882 (9)	0.255 (2)	0.056 (7)*	
C5	0.3380 (4)	0.74759 (7)	0.3472 (2)	0.0377 (5)	
H5	0.406 (4)	0.7714 (7)	0.414 (2)	0.039 (5)*	
C6	0.4694 (4)	0.70252 (8)	0.3461 (2)	0.0369 (5)	
H6	0.647 (5)	0.6940 (8)	0.407 (2)	0.050 (6)*	
C7	0.3643 (4)	0.66740 (7)	0.24958 (19)	0.0298 (4)	
H7	0.461 (4)	0.6359 (8)	0.250 (2)	0.038 (5)*	
C8	0.0147 (4)	0.56249 (6)	−0.05929 (18)	0.0242 (4)	
H8A	0.127 (4)	0.5338 (7)	−0.042 (2)	0.029 (5)*	
H8B	−0.185 (4)	0.5556 (7)	−0.0494 (19)	0.033 (5)*	
C9	0.0378 (3)	0.57542 (6)	−0.20796 (17)	0.0213 (3)	
C10	0.2253 (4)	0.60969 (6)	−0.25333 (19)	0.0276 (4)	
H10	0.344 (4)	0.6278 (7)	−0.188 (2)	0.035 (5)*	
C11	0.2298 (4)	0.61687 (7)	−0.3940 (2)	0.0345 (4)	
H11	0.358 (5)	0.6417 (8)	−0.427 (2)	0.049 (6)*	
C12	0.0502 (4)	0.58950 (8)	−0.4893 (2)	0.0376 (5)	
H12	0.054 (5)	0.5944 (8)	−0.585 (2)	0.048 (6)*	
C13	−0.1299 (4)	0.55588 (7)	−0.44091 (19)	0.0320 (4)	
H13	−0.260 (4)	0.5361 (7)	−0.499 (2)	0.041 (6)*	
Cl1	0.55721 (9)	0.528402 (15)	0.23039 (4)	0.02736 (14)	

### Atomic displacement parameters (Å^2^)

**Table:**

	*U*^11^	*U*^22^	*U*^33^	*U*^12^	*U*^13^	*U*^23^
O1	0.0307 (6)	0.0280 (7)	0.0314 (7)	0.0022 (5)	−0.0081 (5)	−0.0028 (5)
N1	0.0298 (8)	0.0213 (7)	0.0188 (8)	0.0000 (6)	−0.0043 (6)	−0.0013 (6)
N2	0.0251 (7)	0.0234 (7)	0.0235 (8)	0.0004 (6)	−0.0007 (6)	−0.0031 (6)
C1	0.0237 (8)	0.0229 (8)	0.0194 (9)	−0.0026 (6)	0.0012 (6)	0.0006 (7)
C2	0.0278 (8)	0.0222 (8)	0.0203 (9)	−0.0053 (7)	0.0061 (6)	−0.0011 (7)
C3	0.0348 (10)	0.0260 (10)	0.0373 (12)	−0.0017 (8)	0.0005 (8)	−0.0044 (8)
C4	0.0487 (12)	0.0254 (10)	0.0490 (13)	−0.0043 (9)	0.0079 (10)	−0.0115 (9)
C5	0.0449 (11)	0.0366 (11)	0.0323 (11)	−0.0165 (9)	0.0083 (9)	−0.0164 (9)
C6	0.0398 (11)	0.0416 (12)	0.0280 (11)	−0.0110 (9)	−0.0017 (8)	−0.0070 (9)
C7	0.0340 (9)	0.0290 (9)	0.0259 (10)	−0.0049 (8)	0.0009 (7)	−0.0028 (8)
C8	0.0312 (9)	0.0179 (8)	0.0227 (9)	−0.0032 (7)	−0.0001 (7)	−0.0016 (7)
C9	0.0229 (8)	0.0180 (8)	0.0222 (9)	0.0036 (6)	−0.0016 (6)	−0.0028 (7)
C10	0.0290 (9)	0.0253 (9)	0.0278 (10)	−0.0025 (7)	0.0005 (7)	0.0005 (8)
C11	0.0384 (10)	0.0359 (10)	0.0302 (11)	−0.0014 (8)	0.0086 (8)	0.0047 (9)
C12	0.0444 (11)	0.0466 (12)	0.0218 (10)	0.0048 (9)	0.0043 (8)	0.0011 (9)
C13	0.0339 (10)	0.0369 (11)	0.0236 (10)	0.0044 (8)	−0.0040 (7)	−0.0069 (8)
Cl1	0.0294 (2)	0.0264 (2)	0.0248 (2)	0.00059 (16)	−0.00344 (16)	−0.00006 (17)

### Geometric parameters (Å, °)

**Table:**

O1—C1	1.2308 (19)	C5—H5	0.95 (2)
N1—C1	1.345 (2)	C6—C7	1.391 (3)
N1—C8	1.443 (2)	C6—H6	0.99 (2)
N1—H1N1	0.86 (2)	C7—H7	0.97 (2)
N2—C13	1.340 (2)	C8—C9	1.499 (2)
N2—C9	1.345 (2)	C8—H8A	0.946 (19)
N2—H1N2	0.93 (2)	C8—H8B	0.95 (2)
C1—C2	1.499 (2)	C9—C10	1.384 (2)
C2—C3	1.387 (2)	C10—C11	1.380 (3)
C2—C7	1.395 (2)	C10—H10	0.93 (2)
C3—C4	1.384 (3)	C11—C12	1.389 (3)
C3—H3	0.98 (2)	C11—H11	0.98 (2)
C4—C5	1.378 (3)	C12—C13	1.361 (3)
C4—H4	0.94 (2)	C12—H12	0.94 (2)
C5—C6	1.379 (3)	C13—H13	0.95 (2)
			
C1—N1—C8	120.53 (14)	C6—C7—C2	119.81 (18)
C1—N1—H1N1	122.9 (14)	C6—C7—H7	119.5 (11)
C8—N1—H1N1	115.7 (14)	C2—C7—H7	120.7 (11)
C13—N2—C9	123.16 (16)	N1—C8—C9	113.30 (14)
C13—N2—H1N2	119.3 (13)	N1—C8—H8A	108.1 (11)
C9—N2—H1N2	117.6 (13)	C9—C8—H8A	105.5 (12)
O1—C1—N1	121.02 (15)	N1—C8—H8B	111.8 (12)
O1—C1—C2	121.53 (15)	C9—C8—H8B	108.5 (11)
N1—C1—C2	117.43 (14)	H8A—C8—H8B	109.4 (16)
C3—C2—C7	119.00 (16)	N2—C9—C10	118.35 (16)
C3—C2—C1	117.44 (15)	N2—C9—C8	116.02 (14)
C7—C2—C1	123.56 (15)	C10—C9—C8	125.59 (15)
C4—C3—C2	120.69 (18)	C11—C10—C9	119.45 (17)
C4—C3—H3	120.5 (12)	C11—C10—H10	121.3 (12)
C2—C3—H3	118.8 (12)	C9—C10—H10	119.2 (12)
C5—C4—C3	120.15 (19)	C10—C11—C12	120.25 (18)
C5—C4—H4	119.7 (13)	C10—C11—H11	119.8 (13)
C3—C4—H4	120.2 (14)	C12—C11—H11	120.0 (13)
C6—C5—C4	119.87 (18)	C13—C12—C11	118.67 (19)
C6—C5—H5	120.8 (12)	C13—C12—H12	121.2 (13)
C4—C5—H5	119.3 (12)	C11—C12—H12	120.1 (13)
C5—C6—C7	120.47 (19)	N2—C13—C12	120.11 (17)
C5—C6—H6	123.1 (13)	N2—C13—H13	116.0 (13)
C7—C6—H6	116.3 (13)	C12—C13—H13	123.9 (13)

### Hydrogen-bond geometry (Å, °)

**Table:**

*D*—H···*A*	*D*—H	H···*A*	*D*···*A*	*D*—H···*A*
N1—H1N1···Cl1	0.86 (2)	2.41 (2)	3.2057 (15)	153.3 (18)
N2—H1N2···Cl1^i^	0.93 (2)	2.13 (2)	3.0446 (16)	171.7 (18)

Symmetry codes: (i) −*x*, −*y*+1, −*z*.
